# Study on the relationship between nephrotic syndrome and atopic diseases in childhood

**DOI:** 10.3389/fped.2022.992862

**Published:** 2022-10-13

**Authors:** Yue Zheng, Xuehui He, Ling Hou, Xiuli Wang, Chengguang Zhao, Yue Du

**Affiliations:** Department of Pediatrics, Shengjing Hospital of China Medical University, Shenyang, China

**Keywords:** histamine, bradykinin, primary nephrotic syndrome, permeability factor, foot process effacement

## Abstract

**Objective:**

The present study aimed to explore the relationship between nephrotic syndrome and atopic diseases in childhood.

**Methods:**

From 2018 to 2019, 234 children with first-onset primary nephrotic syndrome (PNS) were selected for observation and long-term follow-up, and the clinical and laboratory data. To compare the levels of total serum IgE, histamine and bradykinin of the same children at the time of first onset, remission and relapse of PNS. The extent of podocyte foot process effacement was compared between the urinary protein negative-conversion group and the proteinuric group with the NS range. The correlation between the urine protein quantification and the extent of foot process effacement was also observed.

**Results:**

(1) The mean age of 234 children with first-onset PNS was 4.82 ± 3.63 years, with a male to female ratio of 162/72. (2) There were 109 cases (46.58%) with concomitant atopic diseases (AD) and 151 cases (64.53%) with elevated levels of total serum IgE. There were 136 cases with recurrence during the follow-up, of which recurrence due to allergy-related factors was greater than that due to infection-related factors. (3) The total IgE and bradykinin serum levels were significantly higher in children with first-onset PNS and recurrent PNS compared with those in remission, and the differences were statistically significant (*P* < 0.05). The level of histamine in children with first-onset PNS was higher than that in children with remission (*P* < 0.05), and there was no significant difference in the level of histamine between children in the recurrence group and those in the remission group (*P* > 0.05). (4) There was no significant difference in the extent of foot process effacement between the urinary protein negative-conversion group and the proteinuric group with the NS range. There was no significant correlation between the proteinuria quantification and the extent of foot process effacement.

**Conclusion:**

There existed a high co-morbidity with AD in children with PNS, and allergy-related factors might be an important recurrence factor in children with PNS. The injury to the filtration barrier in MCD might not only be correlated with podocyte lesions but also with some serum permeability factors. Serum IgE, histamine, and bradykinin might be the plasma permeability factors in children with PNS.

## Introduction

The most common pathological type of primary nephrotic syndrome (PNS) in children is minimal change disease (MCD), which is generally sensitive to steroid therapy, undergoes no obvious morphological changes under light microscopy, and shows varying degrees of foot process effacement under electron microscopy. Previous studies have found that some children with negative conversion of urinary protein continue to exhibit the renal pathology of foot process effacement, which is similar to that of the foot process effacement in those with proteinuria within the nephrotic syndrome (NS) range (1). This suggests that MCD may not only be correlated with podocyte lesions. The children with negative conversion of urinary protein after steroid therapy might first be achieved by repairing other links in the filtration barrier other than the foot process, rather than directly repairing the foot process effacement. This can explain the persistence of foot process effacement despite the negative conversion of urine protein. In 1954, Gentili et al. (2) first suggested that some unknown serum circulating factor might be present in patients with idiopathic NS. Shalhoub (3) further suggested that certain circulating factors might affect the glomerular permeability. Since then, in the field of nephrology, researchers have been trying to decipher the identity of the mysterious permeability factors in patients with idiopathic NS, and there have been successive reports on the involvement of permeability factors in the pathogenesis of NS, especially in focal segmental sclerosis nephropathy (4–7). There is no definite conclusion as to whether there is a particular permeability factor present in the blood of children with steroid-sensitive NS (SSNS), although it has been shown that the immunoglobulin E (IgE)-dependent histamine-releasing factor is significantly elevated in NS (8). It has been noted in the literature that montelukast (a cysteinyl leukotriene receptor antagonist) might be an adjunct to the treatment of NS in children (9, 10). The latest study has even suggested that cetirizine (a histamine H1 receptor antagonist) can be used as an adjunctive treatment for adults with MCD and atopic disease (AD) (11), effectively reducing the dose of glucocorticoids and alleviating the side effects of steroids. All of the above findings suggest that chronic allergic inflammation plays a role in the pathogenesis of PNS, but it is inconclusive whether the allergic inflammatory mediators are one of the permeability factors in the plasma in children with NS (12).

Histamine and bradykinin are typical allergic inflammatory mediators, and many studies have reported on respiratory hyperreactivity and cutaneous allergic diseases. Bradykinin regulates inflammatory signaling pathway, enhances the expression of cyclooxygenase-2 (COX-2) and stimulates Prostaglandin E2 (PGE2), thereby promoting the production of proteinuria and podocyte apoptosis (13). There are four homologous G protein-coupled receptors for histamine, which trigger glomerular and tubular degeneration through different histamine receptor pathways. The distribution and effects of the four histamine receptors are different. H1 and H2 receptors are mainly expressed in renal vessels, while H3 and H4 receptors are mainly expressed in renal units and collecting ducts. The effect of histamine on the kidney is mainly related to its vasoactive properties. Under physiological conditions, histamine can act on this organ in an autocrine manner, and under pathological conditions, whether autocrine or paracrine, the renal inducer pool or extra-renal source of histamine (such as mast cells) may be involved. Increased proteinuria and water and salt excretion, as well as decreased creatinine and urea clearance, are mediated primarily by H1 and H4 receptors. In addition, histamine increases vascular permeability through H1R and targets glycocalyx on the GEC surface, glomerular basement membranes, and podocytes, disrupting the integrity of the glomerular filtration barrier (7, 14, 15).

However, there are no definite results on the alterations of histamine and bradykinin during first onset, remission, and relapse in children with SSNS. In the present study, data concerning changes in histamine and bradykinin in children with SSNS at the time of the first onset, remission, and relapse were collected through the long-term follow-up of 234 children with first-onset PNS. The findings clarified that MCD in children might be correlated not only with podocytes but also with the presence of chronic allergic inflammatory mediators in the blood that injure the filtration barrier. These results provide new options to explore and a basis for the treatment of children with PNS.

## Materials and methods

### Study subjects

The present study was a prospective cohort study. Between January 1, 2018, and December 31, 2019, 234 children with first-onset PNS who were effective on steroid hormone induction therapy, regardless of the manifestations of AD, were screened and long-term followed up at the Department of Pediatric Nephrology and Rheumatology, Shengjing Hospital of China Medical University.

### The diagnostic criteria for PNS in children

The criteria for first onset: Proteinuria within the NS range (a urine protein-to-creatinine ratio [UPCR] in early morning urine (EMU) ≥200 mg/mmol [2 mg/mg] or 24-h urine protein ≥1000 mg/m^2^, with a dipstick result for the detection of urine protein of 3+ or 4+) together with hypoalbuminemia (serum albumin <25 g/L) or edema (if the serum albumin data were not available); patients between the age of 1 and 14 years old.

The criteria for remission: At least three consecutive UPCR (EMU or 24-h urine) ≤20 mg/mmol (0.2 mg/mg) or the dipstick for the detection of urine protein demonstrating trace or negative urine protein.

The criteria for recurrence: Recurrence of proteinuria within the NS range in a child with a history of partial or complete remission; urine protein ≥3+ on the dipstick or EMU UPCR ≥200 mg/mmol (2 mg/mg) with or without edema on three consecutive days.

The criteria for low-dose steroid dependence: Children with the simple type of first-onset PNS, who were sensitive to the initial oral prednisone therapy; recurrent relapse occurring with a reduction in the dose of prednisone to 0.25 mg/kg.d every other morning.

The exclusion criteria: ① Patients with secondary and congenital NS; ② patients with combined severe cardiac and hepatic insufficiency; ③ patients with acute infection; ④ patients with incomplete data (16).

### The diagnostic criteria for AD

Diagnostic criteria for allergic rhinitis in children (17): (1) Symptoms: Two or more of the four symptoms of sneezing, clear, watery nasal discharge, nasal itching, and nasal congestion. The symptoms had persisted or accumulated for more than 1 h per day and were possibly accompanied by other concomitant symptoms, such as respiratory symptoms (coughing, wheezing, etc.) and ocular symptoms (including itching, tearing, redness, and a burning sensation in the eyes). (2) Signs: The common signs are pale, edematous nasal mucosa and watery nasal discharge. (3) Laboratory detection: Positive for at least one allergen using a skin prick test (SPT) and/or serum specific IgE in allergen testing. In the nasal secretion test, an eosinophil ratio >0.05 under high magnification microscopy was considered positive. SPT or serum-specific IgE testing was not required, and the diagnosis could be confirmed based on the allergy history, family history, typical symptoms, and signs alone.

Cough variant asthma is a specific type of asthma. Diagnostic criteria for cough variant asthma in children (18): A recurrent or persistent cough for more than 1 month. The onset of coughing was in the early morning or at night and became worse after activity or exposure to cold air or allergens; patients with no clinical symptoms of infection and poor long-term therapeutic results; patients with therapeutic effects under anti-allergy or bronchodilators therapy; patients with the exclusion of other respiratory diseases that lead to a chronic cough.

The diagnostic criteria for atopic dermatitis in children are as follows (19): ① Itching; ② typical form and location (flexual dermatitis) or atypical form and location with xeroderma; ③ chronic or chronic recurrent course of the disease.

### Study methods

The baseline data (age, gender, blood routine test, blood biochemistry,24-h urine protein quantification, etc.) were collected from 234 hospitalized children diagnosed with first-onset PNS who were effective on steroid hormone induction therapy between January 01, 2018, and December 31, 2019, in the Pediatric Rheumatology and Immunology Unit of Shengjing Hospital of China Medical University. All patients were treated with medium and long course of corticosteroid treatment and followed up until December 31, 2020, for the following: recurrence rate; incidence of co-morbidities (including allergic rhinitis, cough variant asthma, atopic dermatitis); concomitant symptoms including infection(fever or symptoms/assistant investigations of respiratory, gastrointestinal, or urinary tract infections), acute attack stage of respiratory allergies (without fever and meeting the diagnosis of cough variant asthma and/or allergic rhinitis); and acute attack stage of cutaneous allergies (swelling after insect bites/urticaria/atopic dermatitis). For some children with recurrent PNS, 2–4 ml of fasting venous blood was collected in the early morning during the periods of first onset, remission, and recurrence and centrifuged at 2,000 r/min for 15 min at 4°C. The supernatant was extracted and stored in a low-temperature refrigerator at −80°C for further analysis. The levels of histamine and bradykinin in the blood were measured according to the instructions in the enzyme-linked immunosorbent assay diagnostic kit.

Nineteen patients underwent renal pathology biopsy with informed consent from the family due to low-dose hormone dependence or frequent relapses. Renal pathology analysis was conducted at the time of negative conversion of urine protein in 10 patients and proteinuria within the NS range in 9 patients. The degree of foot process effacement under electron microscopy was observed between the two groups, and correlation analysis was conducted between the extent of foot process effacement and urine protein quantification.

The present trial was approved by the Ethics Committee of Shengjing Hospital Affiliated to China Medical University (ethics number: 2019PS096J). This study was conducted in accordance with the declaration of Helsinki. Written informed consent was obtained from all guardians of the patient.

### Statistical analysis

SPSS 24.0 software was adopted for the statistical analysis. For the measurement data that satisfied normal distribution following a single sample K–S test, means ± standard deviation (x ± s) was used to present the results. Countable data were expressed as a percentage (%), and the paired t-test was used for a comparison of measurement data between the groups. The χ2 test was adopted for the comparison between countable data. A Pearson correlation analysis was used for the analysis of the correlation between the area of foot process effacement and urine protein quantification. *P* < 0.05 was considered statistically significant.

## Results

### The general characteristics of children with first-onset PNS

In the present study, there were 234 cases of children with first-onset PNS, with an average age of 4.82 ± 3.63 years. The male-to-female ratio was 162/72, urine protein quantification was 4.12 ± 3.78 g/24 h, serum albumin was 17.0 ± 3.76 mmol/L, and serum cholesterol was 10.07 ± 2.52 mmol/L. These measurements met the diagnostic criteria for PNS.

### The co-morbidity characteristics of children with PNS and AD

As shown in Table 1, the results of the present study identified 109 cases (46.58%) of co-morbidity with AD in 234 children with PNS. Of these, there were 68 cases (29.06%) with a respiratory allergy, 15 cases (6.41%) with allergic rhinitis, 53 cases (22.65%) with cough variant asthma, and 41 cases (17.52%) with atopic dermatitis. The prevalence were higher than the 17% of allergic asthma and 12.94% of atopic dermatitis in the normal population (20, 21). In addition, it was found that significantly elevated levels of total serum IgE were present in 151 cases (64.52%). These findings suggest that there is high co-morbidity between PNS and AD.

**Table 1:** The co-morbidity in children with PNS and atopic diseases and the triggers of recurrence and season of recurrence in children with PNS.

				**N (%)**	***P***
The first onset (n = 234)	Respiratory co-morbidity※			68 (29.06%)	
	Allergic rhinitis			15 (6.41%)	
	Cough variant asthma			53 (22.65%)	
	Cutaneous co-morbidity※			41 (17.52%)	
	Atopic dermatitis			41 (17.52%)	
	Increased total IgE※			151 (64.53%)	
The recurrence (*n* = 136)	Concomitant symptoms in recurrence	Infection		36 (26.47%)	< 0.001
		Allergy-related factor	Respiratory allergy	70 (51.47%)	
			Cutaneous allergy	30 (22.06%)	

### Analysis of triggers of recurrence in children with recurrent PNS

In the present study, 234 children with PNS were followed up for a minimum of 7 months and a maximum of 18 months. Of these, 136 patients had recurrent NS, 91 had no recurrence, and seven were lost to follow-up. Factors related to allergies and infection that might be correlated with the recurrence of NS were studied separately. The results showed that of the 136 children with recurrent PNS, 36 (26.47%) had recurrence accompanied by infection, 70 (51.47%) by a respiratory allergy, and 30 (22.06%) by a cutaneous allergy. These results suggest that the recurrence of NS caused by allergy-related factors in children with PNS is significantly higher than that caused by infection-related factors (P < 0.05) (as shown in Table 1).

### Analysis of the changes in the levels of total IgE, histamine, and bradykinin in children with PNS at different times

We performed a log transformation on the serum IgE data. The intention to do a log transformation was to reduce the variability of IgE data and make the data closer to the normal distribution due to the large and discrete distribution of IgE data. The levels of total serum IgE in 136 children and the levels of histamine and bradykinin in 25 children at the time of first onset, remission, and relapse were measured. The results indicate that the levels of total serum IgE and bradykinin were significantly higher (*P* < 0.05) in children with first-onset PNS and recurrent PNS compared with those in remission from PNS (as shown in Table 2), and the differences were statistically significant. Although the levels of histamine in children in the first-onset group were higher than those in the remission group (*P* < 0.05), the levels of histamine in children in the recurrent group were not significantly different from those in the remission group (*P* > 0.05).

**Table 2:** The changes in the serum total IgE, histamine and bradykinin in children with PNS at the first onset, remission and relapse groups.

**Item**	**Serum total IgE (*****n*** = **136)**		**Serum histamine (ng/ml)**	**Serum bradykinin (ng/ml)**
	**The original value (IU/ml)**	**The log-transferred value**	**(*n* = 25)**	**(*n* = 25)**
The first onset group	688.38 ± 815.90	2.41 ± 0.71	28.61 ± 28.79	1234,21 ± 1247.31
The remission group	250.27 ± 411.15	1.91 ± 0.70	9.02 ± 5.28	595.69 ± 387.47
The recurrence group	699.71 ± 894.27	2.32 ± 0.75	9.72 ± 7.84	973.95 ± 469.9
F	26.496	15.302	14.724	7.010
P	0.000	0.000	0.001	0.030

### Comparative analysis of the extent of foot process effacement under electron microscopy after the negative conversion of urinary protein and the extent of foot process effacement in those with proteinuria within the NS range

**Figure 1:**
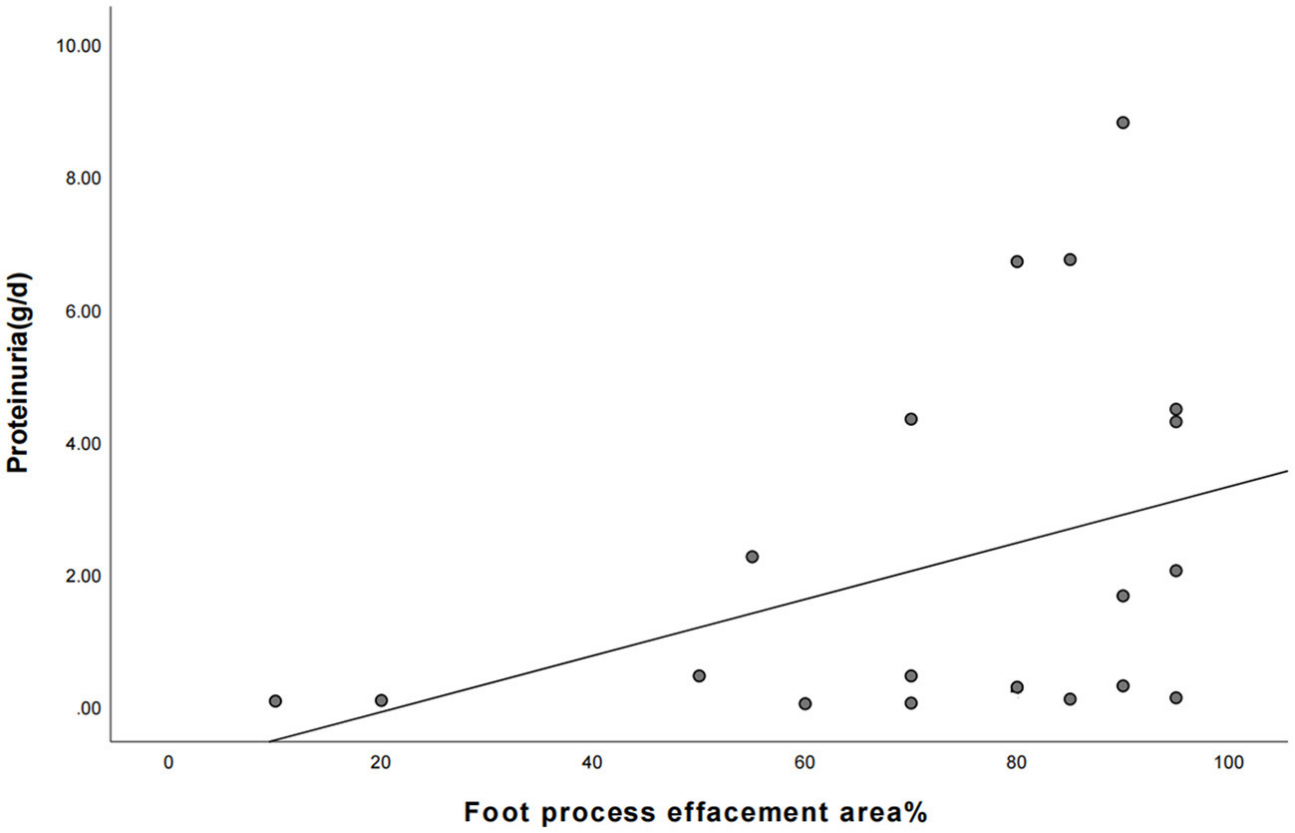
The correlation between the area of foot process effacement and urine protein quantification between the two groups of children (the correlation coefficient r = 0.435, *P* = 0.055).

**Figure 2:**
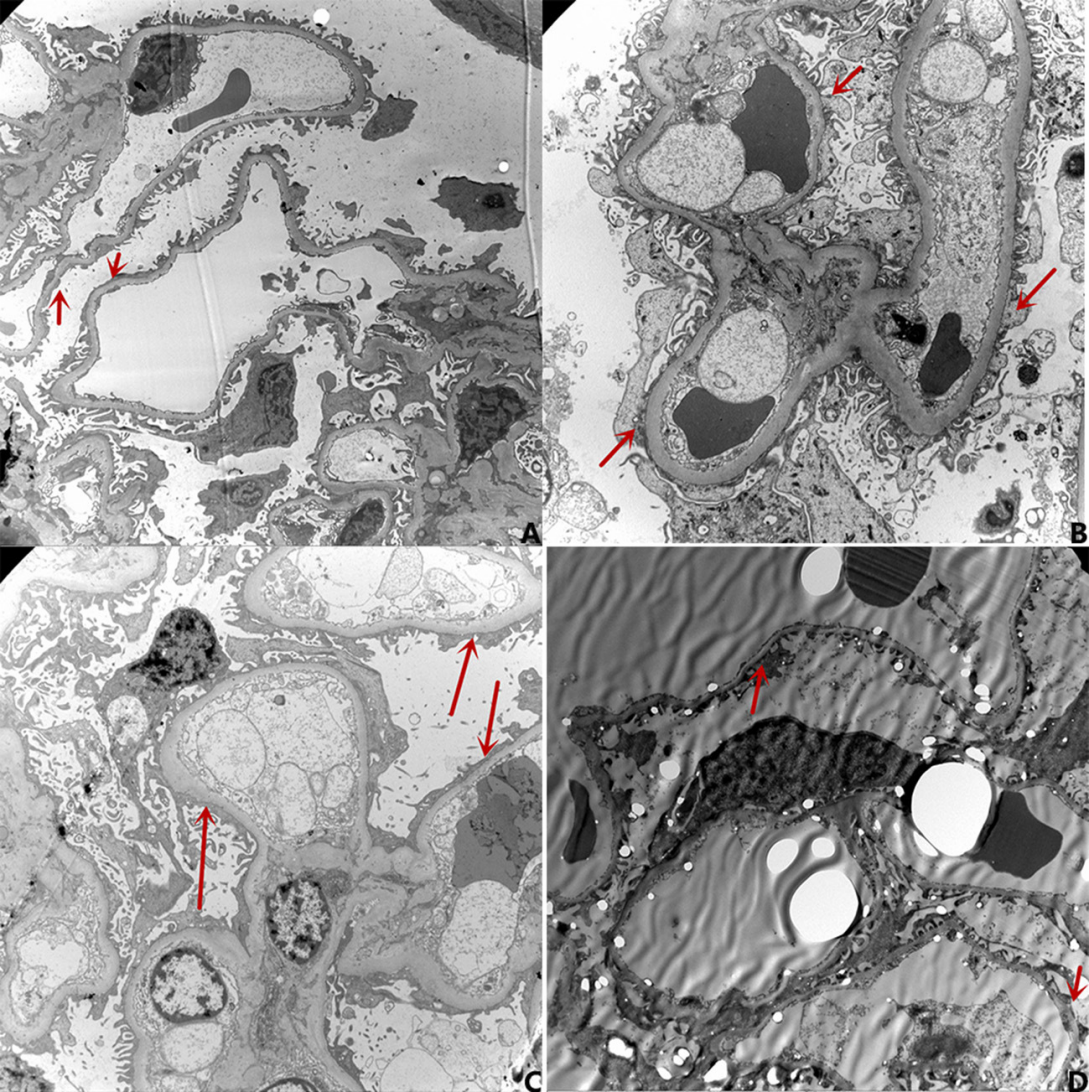
The results of electron microscopy. **(A)** Urine protein: 4.28g/d, Degree of foot process effacement: 95%; **(B)** Urine protein 0.09 g/d (with negative conversion for 4 days), Degree of foot process effacement: 85%; **(C)** Urine protein 0.15 g/d(with negative conversion for 7 days), Degree of foot process effacement: 80%; **(D)** Urine protein 0.01 g/d(with negative conversion for 90 days), Degree of foot process effacement: 10%.

Nineteen patients underwent renal pathology biopsy with informed consent from the family due to low-dose hormone dependence or frequent relapses. Renal pathology analysis was conducted at the time of negative conversion of urine protein in 10 patients and proteinuria within the NS range in nine patients. All renal pathology under electron microscopy still showed MCD and varying degrees of foot process effacement. In one of these patients, urine protein had been negative for 3 months, but foot process effacement was still visible in 10% of the podocytes. In the remaining cases, urine protein was negative for 2–20 days, while foot process effacement was still present, which was similar to the changes in the degree of foot process effacement in children with proteinuria within the NS range. There was no significant difference in the extent of foot process effacement between the children in the urinary protein negative-conversion group and the proteinuric group within the NS range (*P* > 0.05). Moreover, a correlation analysis showed that there was no significant correlation between the proteinuria quantification and area of foot process effacement (r = 0.435, *P* = 0.055). The detailed data are set out in Table 3 and Figure 1, and the results of electron microscopy are shown in Figure 2.

**Table 3:** The comparison of the area of foot process effacement and urine protein quantification between the urine protein negative conversion group and the urine protein positive group.

**Group**	**Number of cases**	**The area of foot process effacement (%)**	**The urine protein quantification (g/d)**
The urine protein negative conversion group	10	70.00 ± 22.24	0.1960 ± 0.1586
The urine protein positive group	9	83.89 ± 13.64	4.5800 ± 2.4408
Z	-	−1.650	−3.676
P	-	0.099	<0.001

## Discussion

The most common pathological type of PNS in children is MCD. There are no obvious morphological changes under light microscopy, although different degrees of foot process effacement are visible under electron microscopy. Previous studies on the electron microscopic alterations in NS (22) have shown that in addition to foot process effacement, changes in the light and dense layers of the basement membrane are visible in children with NS under electron microscopy. A recent study on such changes (23) has demonstrated that in addition to changes in podocytes, the disappearance of endothelial cell fenestrations and the disappearance of the cytoplasmic honeycomb sieve structure of endothelial cells is also visible, and it is believed that patients with changes in endothelial cells might have a poor prognosis. Bauer et al. (24) suggested that alterations involving the systemic and glomerular endothelium are nearly universal in patients with MCD, and that Glomerular endothelial cell can be directly activated by circulating factors present in the MCD sera during relapse. The above literature suggests that in terms of the pathological changes in kidneys in NS, in addition to foot process effacement, changes in endothelial cells and the basement membrane may also be involved in the pathogenesis and process of NS. Moreover, the main structures of the filtration barrier include the glycocalyx, endothelial cells, the basement membrane, and podocytes. They all might be involved in the pathogenesis of NS, while the morphological changes mainly manifest as foot process effacement, and endothelial cells and the basement membrane only manifest as functional abnormalities without obvious morphological changes. Ten patients of negative conversion of urine protein underwent renal pathology biopsy in our study. In one of these children, urine protein had been negative for 3 months, but foot process effacement was still visible in 10% of the podocytes. In the remaining cases, urine protein was negative for 2–20 days, although foot process effacement was still present, which was similar to the changes in the degree of foot process effacement in children with proteinuria within the NS range. There was no obvious correlation between the proteinuria quantification and the severity of foot process effacement, which is similar to previous results (1, 25). The negative conversion of urinary protein after steroid therapy might first be attained by repairing other links in the filtration barrier other than the foot process effacement. This could be achieved, for example, by reducing the permeability factor in the plasma or by repairing the function (or structure) of the glycocalyx, endothelium, and/or basement membrane rather than by directly repairing the foot process effacement. With the improvement in proteinuria, the morphological changes in foot process effacement would gradually be repaired. This could explain the phenomenon of negative urine protein with persistent foot process effacement.

A substance in the serum of patients with NS exists that might be involved in the development of NS. In 1957, Gentili et al. infused the plasma from patients with NS into healthy subjects, resulting in the occurrence of temporary proteinuria in these subjects, and proposed for the first time that there might exist glomerular permeability factors in the plasma of patients with NS. Subsequent studies have further confirmed the presence of permeability factors in patients with NS. Hoyer observed the recurrence of NS after renal transplantation in patients with steroid-resistant NS (26), while Zimmerman injected serum from patients with focal segmental glomerulosclerosis (FSGS) into healthy rats, which rapidly produced proteinuria (27). Patients with NS have shown a rapid reduction in urinary protein after plasma replacement or immunosorbence (28, 29). When the kidney from a patient with FSGS was transplanted into another recipient with end-stage renal disease due to diabetic nephropathy, a rapid decrease in proteinuria and the disappearance of histological lesions was observed by biopsy (30). In addition, clinical studies have shown that rituximab is effective in both membranous nephropathy and steroid-dependent NS, such as MCD, indicating that there might be a similar pathogenesis of these two pathological changes. Studies have demonstrated that membrane nephropathy is caused by the production of antiphospholipase A2 receptor antibodies by plasma cells, leading to the assumption that the pathogenesis of MCD might also be the excessive production of a substance by plasma cells. Therefore, some kind of permeability factor in the plasma might lead to the increased permeability of the filtration barrier. To date, there is no definitive answer as to what exactly the plasma permeability factor is.

In the present study, it was found that 64.53% of children with PNS had higher total serum IgE levels than healthy children of the same age and 46.58% had co-morbidity with AD, which is consistent with the results of previous studies (31). Through the investigation of the concomitant symptoms in patients with recurrent PNS, it was found that the triggers of PNS recurrence in children, allergy-related factors are more significant than infection-related factors. Therefore, it was hypothesized in the present study that the onset and recurrence of PNS in children might be closely correlated with allergy-related factors, suggesting that allergic inflammatory mediators might be involved in the pathogenesis of PNS in children, possibly as one of the unknown permeability factors in the blood. Previous studies have shown that histamine and bradykinin are important inflammatory mediators in AD, and children with PNS showed high co-morbidity with AD. We indicate that histamine and bradykinin might be involved in the pathogenesis of PNS. The levels of total serum IgE in 136 children and the levels of histamine and bradykinin in 25 children at the time of first onset, remission, and relapse were measured. The results demonstrated that the levels of total IgE and bradykinin were significantly higher in children with first-onset PNS and recurrent PNS than in those in the remission group, and the differences were statistically significant. Although the level of histamine in children in the first-onset PNS group was higher than those in the remission group, the difference in the levels of histamine was not statistically significant between children in the recurrent group and those in the remission group. The results of the present study suggest that there were significant differences in the levels of serum IgE and bradykinin in children with PNS at first onset, remission, and relapse and in the levels of histamine at first onset and remission, suggesting that allergic inflammatory mediators in the plasma of children with PNS might be involved in the pathogenesis of NS in children. These results also reaffirmed the possible presence of some circulating permeability factors in the blood of children with PNS and that the levels of IgE, histamine, and bradykinin might be among the circulating permeability factors in the blood of these children.

## Conclusion

In summary, the pathogenesis of MCD-type NS in children might be characterized by increased permeability of the filtration barrier, resulting in the permeation of albumin from the blood into the urine, and foot process effacement might act as a pathological basis, but not the only one. MCD might not only be correlated with podocyte lesions but also with certain permeability factors in the blood as well as changes in the renal vascular endothelium and basement membrane. PNS in children showed a high co-morbidity with AD. In cases with recurrence of PNS in children, the number of cases with allergy-related factors as the trigger was significantly higher than the number of cases triggered by infection-related factors. This suggests that PNS and AD are the same category of disease with a similar pathogenesis and a reflection of the same pathological process in different organs. The differences in the levels of allergic inflammatory mediators, including IgE, histamine, and bradykinin, were statistically significant among children with PNS at the first onset, remission, and recurrence, which might indicate that plasma permeability factors are involved in the onset and relapse of PNS in children. The above findings can provide new options to explore and a basis for the treatment of children with PNS.

## Data availability statement

The original contributions presented in the study are included in the article/supplementary material, further inquiries can be directed to the corresponding author/s.

## Ethics statement

The studies involving human participants were reviewed and approved by Ethics Committee of Shengjing Hospital Affiliated to China Medical University (Ethics Number: 2019PS096J). Written informed consent to participate in this study was provided by the participants' legal guardian/next of kin.

## Author contributions

YZ conceived the idea and conceptualized the study. XH and LH collected the data and analyzed the data. XW and CZ drafted the manuscript. YZ and YD reviewed the manuscript. All authors read and approved the final draft.

## Conflict of interest

The authors declare that the research was conducted in the absence of any commercial or financial relationships that could be construed as a potential conflict of interest.

## Publisher's note

All claims expressed in this article are solely those of the authors and do not necessarily represent those of their affiliated organizations, or those of the publisher, the editors and the reviewers. Any product that may be evaluated in this article, or claim that may be made by its manufacturer, is not guaranteed or endorsed by the publisher.
